# Moral distress among inpatient child and adolescent psychiatry staff: a mixed-methods study of experiences and associated factors

**DOI:** 10.1186/s13034-025-00868-7

**Published:** 2025-02-28

**Authors:** Nora Eder, Kristin Nordenberg, Niklas Långström, Alexander Rozental, Astrid Moell

**Affiliations:** 1 Stockholm Centre for Eating Disorders, Region Stockholm, Stockholm, Sweden; 2 Child and Adolescent Psychiatric Services, Region Stockholm, Stockholm, Sweden; 3 Centre for Psychiatry Research, Department of Clinical Neuroscience, Karolinska Institutet, & Stockholm Health Care Services, Region Stockholm, Stockholm, Sweden; 4 Department of Health, Education and Technology, Luleå University of Technology, Luleå, Sweden

**Keywords:** Child and adolescent mental health, Psychiatry, Moral distress, Coercive measures, Health care professionals, Inpatient care

## Abstract

**Background:**

Moral distress among healthcare staff is associated with emotional strain and workforce attrition but remains insufficiently explored in child and adolescent psychiatry (CAP). We investigated the experiences and factors contributing to moral distress among staff in inpatient CAP.

**Methods:**

We conducted a 2023 nationwide mixed-methods survey with 106 staff members from CAP inpatient units in Sweden. Quantitative data on moral distress were collected using the Stress of Conscience Questionnaire, while open-ended questions provided qualitative data on perceptions of moral distress and the impact of working with coercive measures. Findings were integrated using triangulation.

**Results:**

Staff reported high levels of moral distress, with physicians experiencing the highest. Triangulation revealed converging findings with younger and less experienced staff experiencing more moral distress. Aligning with the qualitative theme “Providing care one does not believe in”, moral distress was also strongly associated with quantitative data of a demanding work environment, low social support, and intention to resign from job. The theme “Ethical dilemmas about coercive measures” indicated how coercive measure use could contribute to moral distress, while a more positive attitude to coercive measures was associated with lower moral distress.

**Conclusions:**

Moral distress among CAP inpatient staff in Sweden was pronounced, particularly among younger, less experienced professionals, and physicians. Addressing moral distress appears critical in supporting staff well-being, promoting workforce retention, and maintaining high-quality patient care.

**Supplementary Information:**

The online version contains supplementary material available at 10.1186/s13034-025-00868-7.

## Background

Moral distress, first defined by Jameton [1], describes the psychological discomfort experienced when professionals recognise an ethically appropriate action but feel constrained from taking this action due to institutional limitations. While some ethical discomfort may promote reflection and professional growth [2], sustained moral distress has been linked to emotional exhaustion, burnout, and diminished quality of care [3, 4]. Further, moral distress has been associated with intentions to resign [5, 6]; one study found that 15% of nurses had left a previous position specifically due to moral distress [2].

Given high job demands, organisational limitations, and frequent ethical dilemmas—particularly regarding coercion and patient autonomy—psychiatric professionals may be especially vulnerable to moral distress [4, 7, 8]. For example, among nurses in acute psychiatric care, moral distress has been associated with burnout, job dissatisfaction, cynicism, own mental health issues, and a poorer moral climate at work [8]. The use of coercive measures (e.g., restraining the patient to a belt bed or secluding him/her in a locked room) is often perceived as a negative experience by staff [9], although attitudes tend to vary between professionals [10]. Psychiatric staff have also described various ethical dilemmas associated with coercion; including feelings of powerlessness, loyalty conflicts, and a perceived decline in care quality related to organisational limitations [11–13]. While coercive measures are prevalent in adult psychiatry [14], their use may be more common in child and adolescent psychiatry (CAP) [15]. The more frequent use suggests a potentially greater emotional and ethical burden on psychiatric staff working with children and adolescents.

The current evidence on moral distress in inpatient CAP is limited to qualitative studies. Factors that reportedly contribute to moral distress include ethical dilemmas related to safety, competence, and autonomy [16] as well as conflicts between organisational demands for control, such as coercive measures, and personal commitment to empathy and compassion [12]. Thus, despite growing recognition of moral distress in healthcare professionals, levels and contributing factors to such distress remain underexplored in inpatient CAP.

To address this gap, we conducted the first mixed-methods study from a CAP setting with quantitative and qualitative data on moral distress. Specifically, the study objectives were to: (a) determine the level of moral distress among staff in inpatient CAP across Sweden; (b) examine associations between moral distress and demographic factors (age, gender), professional characteristics (work experience, occupation, region of work), and work environment (intention to resign, psychosocial work environment); (c) analyse the relationship between moral distress and coercive measures, addressing attitudes towards coercion, potential value incongruence, and perceived frequency of use; and (d) qualitatively explore staff experiences of moral distress and their perspectives on working with coercive measures.

## Methods

We conducted a contemporary digital survey to measure moral distress among staff working in CAP inpatient units throughout Sweden. Using a convergent mixed-methods design [17], we collected both quantitative and qualitative data to achieve a more nuanced understanding of moral distress. For details on Swedish CAP inpatient settings, see the supplementary materials of our previous publication [18]. Reporting followed the STROBE Statement [19] and COREQ checklist [20].

### Recruitment and data collection

We employed a gatekeeper-assisted recruitment strategy and contacted heads of all 23 CAP inpatient care units in Sweden asking them to share the survey with their staff via email. To complement this, printed materials with QR codes were distributed through regular mail to units, and the survey was also shared in a professional social media group frequented by CAP professionals in Sweden.

Data were collected through REDCap—a secure, electronic data capture platform, designed for research purposes [21]—using an anonymous online survey (available in the Appendix). To evaluate the survey’s feasibility and completion time, we conducted a pilot test with 22 final-year psychology students (whose responses were not included in the dataset). Data collection spanned seven weeks from January to March 2023. During this period, the heads of all CAP inpatient care units were reminded via calls and emails (one to three times, depending on the regional response rate) to distribute the survey. To ensure anonymity, no personal identification details were collected from participants and data analysis and reporting are done at an aggregate level.

### Measures

We collected data on participants’ gender, age, occupation, region of work, and duration of their professional experience within CAP inpatient care.

To measure the primary outcome of moral distress, we used the *Stress of Conscience Questionnaire (SCQ)*. The SCQ, validated within the Swedish context, was designed for ethical dilemmas specific to healthcare settings [22]. The questionnaire consists of nine items, each with two questions: one addressing the *frequency* of nine different potentially morally distressing situations, and the other the *level of moral distress* felt in each situation. Questions are rated on a six-point Likert scale (0–5), with the scores multiplied within each item and summed to yield a total score of 0–225 [23]. The SCQ has shown sufficient internal consistency with a Cronbach’s alpha of 0.83 [22], confirmed by further international studies [24, 25]. We used SCQ as a unidimensional measure, given recent research suggesting a better fit than the original two-dimensional model [24].

We used the *Staff Attitude to Coercion Scale* (SACS) to assess staff attitudes towards coercive measure use. The SACS comprises 15 items, each rated on a five-point Likert scale (1–5), with higher scores indicating a more positive attitude to coercive measures [26]. Total scores were calculated as a mean ranging 1–5. The scale has been proposed either as one- or three-dimensional, in the latter case with subscales *Coercion as Care and Security* (viewing coercion as necessary for safety and well-being), *Coercion as Offending* (perceiving coercion as ethically troubling or harmful), and *Coercion as Treatment* (considering coercion as a therapeutic intervention). We calculated both one-dimensional and three-dimensional correlations with SCQ scores. A systematic review indicates that the SACS has adequate internal consistency and structural validity (i.e. scale scores reflect construct dimensionality), though some psychometric properties remain unexplored [27].

Participants completed the SACS twice: first, expressing personal views, and second, reflecting perceived organisational views on coercive measures. To quantify *value incongruency* regarding coercive measures, we calculated the absolute difference between each participant’s personal SACS scores and their perceived organisational scores, followed by averaging across all items. This approach, to our knowledge, has not been applied before and represents a novel method for assessing value incongruency in staff attitudes to coercion.

Since we did not have access to rates of coercive measures per unit, we estimated *frequency of coercive measure use* with the question “How frequent is the use of coercive measures at your inpatient ward?”. Responses were provided on a six-point Likert scale: “Every day”, “Often (at least once a week)”, “Sometimes (a few times a month)”, “Rarely (a few times a year)”, “Very rarely (once a year)”, or “Never”.

Psychosocial work environment was measured with the *Demand-Control-Support Questionnaire* (DCSQ). The DCSQ consists of 17 items, divided into three dimensions: job demands, job control, and social support [28]. The questionnaire has been used and validated in several studies and different countries [28, 29].

Participants’* intention to resign from the current job* was assessed by asking, “How often have you considered changing jobs in the past 12 months?”, with options: “Every day”, “Often (at least once a week)”, “Sometimes (a few times a month)”, “Rarely (a few times a year)”, “Very rarely (once a year)”, and “Never”.

Finally, we collected *qualitative data* with three open-ended questions: “What in your work leads to moral distress?”, “How does working with coercive measures affect you?” and “Is there anything else you would like to add?”.

### Participants

We included staff who were clinically active in inpatient CAP at the time of the survey, with no regard to occupational category or form of employment. Of the 121 respondents who consented and initiated the survey, 15 were excluded for providing only demographic data, leaving 106 respondents as the final analytic sample: 28 nurses, 19 psychiatrists/physicians, 42 psychiatric aides, 8 social workers, 4 treatment providers, and 5 who identified as “other staff.” Participants’ mean age was 41.9 years (SD = 11.7), 73% identified as women, and 51% worked in larger metropolitan regions. Further details on participants are presented in Table 1.

**Table 1:** Stress of Conscience Questionnaire scores among professionals in Swedish inpatient child and adolescent psychiatry

	n (%)	Median (IQR)	Correlation/difference (95% CI)	Cohen’s *d*
Total SCQ score	106 (100%)	60.0 (31.0, 86.0)	NA	NA
Gender				
Male	28 (26%)	61.5 (37.0, 89.3)	Ref.	Ref.
Female	77 (73%)	59.0 (30.3, 85.0)	− 3 (− 21, 15)^a^	− 0.09
Non-binary	1 (1%)	86.0 (NA)	NA	NA
Age, yrs, *M* (SD)	41.9 (11.7)		− 0.31***^b^	− 0.65
Professional experience of CAP inpatient care				
≤ 5 years	70 (66%)	66.5 (41.5, 91.8)	Ref.	Ref.
> 5 years	36 (34%)	45.5 (20.8, 68.0)	− 22 (− 36, − 6)**^a^	− 0.67
Occupation				
Physician	19 (18%)	83.0 (64.5, 99.0)		
Nurse	29 (27%)	57.0 (36.1, 86.0)	0.11**^c^	
Social worker or treatment provider	12 (11%)	73.5 (38.0, 117.0)		
Psychiatric aide	46 (43%)	45.5 (28.0, 71.0)		
Work environment (DSCQ)				
Job demands			0.55***^b^	1.32
Job control			− 0.24*^b^	− 0.49
Social support			− 0.44***^b^	− 0.97
Staff attitude to coercion (SACS)				
Total score			− 0.31**^b^	− 0.65
Coercion as offending			0.44***^b^	− 0.97
Coercion as care & security			− 0.20*^b^	− 0.41
Coercion as treatment			0.03^b^	− 0.06
Value incongruency, SACS-based^d^			0.08^b^	0.17
Frequency coercive measures in ward^e^			0.22*^b^	0.45
Intention to resign^f^				
Yes	50 (56%)	79.0 (60.3, 101.8)	Ref.	Ref.
No	40 (44%)	32.5 (16.8, 59.0)	− 44 (− 57, − 30)***^a^	− 1.48

*SCQ *Stress of Conscience Questionnaire, *IQR* Interquartile range, *CI* confidence interval, *NA* not applicable, *M* mean, *SD* standard deviation, *CAP* child and adolescent psychiatry, *DSCQ* Demand Support Control Questionnaire *SACS* Staff Attitude to Coercion Scale

Effect sizes: small: *d* = 0.2–0.49, medium: *d* = 0.5– 0.79, large: *d* = 0.80 +

Missing data: n = 90 for DSCQ, Value incongruency, Intention to resign from job, n = 91 SACS, Frequency coercive measures in ward

^a^Hodges-Lehman estimator [estimated median difference]

^b^Spearman rank correlation coefficient

^c^η^2^, p-value from Kruskal Wallis test

^d^Mean absolute difference in SACS perceived organisational and personal score

^e^Participant-reported ward use of coercive measures

^f^Yes: contemplating it a few times a month—every day, No: a few times a year—never. See also Table 2

*p < 0.05, **p < 0.01, ***p < 0.001, p-values were not calculated for Cohen’s *d*

### Data reduction

For gender-based analyses, the participant identifying as non-binary was excluded due to the small sample size, limiting this analysis to participants identifying as male or female. We created a binary variable for professional experience: working in inpatient CAP for five years or less, or more than five years. Intention to resign from job was also dichotomised: “yes” for those considering resigning every day—a few times a month, and “no” for those considering resigning less often. We clustered occupations into four categories informed by predominant roles in our sample: physicians (a single category including board-certified child and adolescent psychiatrists, adult psychiatrists, residents, or interns), nurses, social workers or treatment providers, and psychiatric aides. Participants with other occupations were categorised by their education level and function in care and assigned to the most appropriate of the four categories. We deemed analyses on possible regional differences unsuitable given notable response rate variations and low statistical power for subsample comparisons.

### Statistical analysis

All analyses were performed in R (version 4.3.1, 2023-06-16), with a two-tailed significance level set at p < 0.05. We used non-parametric methods where appropriate, since data were not normally distributed following the Shapiro–Wilk test and visual inspection of density plots. Differences in moral distress between two-tiered groups (i.e., gender, intention to resign, work experience) were analysed with the Mann–Whitney U test and the Hodges-Lehmann estimator providing robust estimates of median differences between groups and 95% confidence intervals. Associations between moral distress and continuous or ordinal variables (i.e., age, attitude to coercion, difference in attitude to coercion, perceived frequency of coercive measures, and dimensions of the DCSQ: job demands, job control, and social support) were analysed using Spearman rank correlation coefficients. Differences in moral distress across occupation categories were examined using the Kruskal–Wallis test (reported as η^2^), with post hoc pairwise comparisons using the Mann–Whitney U test, adjusted for multiple comparisons using Holm’s method. Hodges-Lehmann estimators with 95% confidence intervals were reported for all pairwise comparisons. Spearman rank correlation coefficients and Hodges-Lehmann estimators were transformed into Cohen’s *d* and interpreted following Cohen’s guidelines [30]: small (*d* = 0.2), medium (*d* = 0.5), large (*d* = 0.8). Visualisations were used to supplement statistical analysis and aid interpretation (see Appendix).

### Missing data

Three participants with missing responses on the primary outcome measure (moral distress measured by the SCQ) had their missing values imputed using the mean of the respective SCQ items for all other respondents, resulting in the final analytical sample of 106 participants. Missing data in the remaining dataset were handled using pairwise deletion unless otherwise specified. Comparisons using t-tests and Fisher’s exact tests revealed no significant differences in demographic variables or the primary outcome between respondents with and without missing data (see Appendix). Therefore, albeit with limited power, we found no evidence to reject the assumption that data were missing at random.

### Qualitative analysis

Out of 106 participants, 54 responded also to the optional open-ended survey questions, producing a qualitative dataset of 4167 words. Data were analysed in Microsoft Excel using reflexive thematic analysis [31, 32], chosen due to its adaptability in areas with limited research. Two female final-year psychology students (NE and KN), now psychologists, conducted the initial analysis under the supervision of AR and AM as part of their master’s thesis. NE and KN had prior experience working in inpatient care for eating disorders and CAP inpatient care, respectively. AR is a male clinical psychologist with experience in adult psychiatry, AM is a female child and adolescent psychiatrist working in CAP inpatient care. We assessed potential biases and preconceptions through ongoing reflexive discussions, critically examining our interpretations and reflecting on how our backgrounds might have influenced the analysis.

Following Braun and Clarke’s suggested six-phase model, we initially (1) familiarised ourselves with the data, (2) generated initial codes, (3) searched for themes, (4) reviewed these themes, (5) defined and named themes, and finally (6) produced the results. NE and KN separately conducted the first four phases of analysis to ensure analytical rigour and minimise bias. After each phase, their findings were compared and consensus reached before proceeding. Phases three, four, five, and six were collaborative efforts, with supervisory input ensuring the quality of theme definitions and data alignment. Translations of quotes from Swedish to English were occasionally slightly modified for clarity. To maintain anonymity, common Swedish names were used as pseudonyms irrespective of participant gender identity. For more details on the construction of themes and additional details of the qualitative analysis, see Appendix.

### Triangulation of data

We employed concurrent methodological triangulation [33], merging both quantitative and qualitative insights. Narrative integration was done by cross-referencing between findings to find similarities and discrepancies between the results, presented with joint display integration. The rationale for this integration was twofold: while quantitative data pinpointed objective measures and correlates of moral distress, qualitative data illuminated subjective experiences and nuances.

## Results

### Quantitative results

The overall mean SCQ score was 61.8 (SD = 36.9, range 1–150, possible range 0–225), see Table 1 for median, IQR and effect sizes.

Moral distress decreased moderately with participant age (*d* = − 0.65), and those with over five years of experience in inpatient child and adolescent psychiatry reported moderately lower distress levels than those with less experience (*d* = − 0.67).

We found significant differences in moral distress levels between occupational groups. Post hoc pairwise comparisons identified that physicians reported significantly higher moral distress compared to psychiatric aides (Hodges-Lehmann estimator: 35, 95% CI: 17, 52, adjusted *p* = 0.003), representing a large effect (*d* = 1.10). Physicians also reported higher levels of moral distress compared to nurses representing a large effect size (d = 0.84), although only borderline significant (Hodges-Lehmann estimator: 27, 95% CI: 6, 47, adjusted *p* = 0.058). No meaningful differences were found between other occupational groups (see Appendix for all results and density plots per occupation).

Work environment substantially impacted moral distress. Higher moral distress was strongly positively linked to demanding job environments (*d* = 1.32) and low social support (*d* = − 0.97), while low job control had a smaller effect (*d* = − 0.49). More than half of the participants considered resigning regularly, see Table 2. Those not considering resigning had markedly lower SCQ scores (median difference = − 44, 95% CI: − 57, − 30), reflecting a large effect size (*d* = − 1.48).

**Table 2:** Staff considering resigning during the last 12 months in inpatient child and adolescent psychiatry

	n (%)
Sometimes (a few times a month)	24 (27%)
Often (at least once a week)	21 (23%)
Every day	5 (6%)
*Sum: yes*	*50 (56%)*
Never	13 (14%)
Very rarely (once a year)	14 (16%)
Rarely (a few times a year)	13 (14%)
*Sum: no*	*40 (44%)*

A more positive attitude overall toward coercive measures on the SACS was moderately strongly associated with lower levels of moral distress (*d* = − 0.65). The subscale *Coercion as Offending* showed a negative correlation with moral distress (*d* = − 0.97), indicating that perceiving coercive measures as less offensive was strongly associated with lower moral distress. *Coercion as Care and Security* showed a weaker negative correlation (*d* = − 0.41), while *Coercion as Treatment* exhibited no practically meaningful association with moral distress (*d* = − 0.06). The frequency of coercive measures in the workplace was weakly positively correlated with moral distress **(***d* = 0.45). Last, value incongruency—differences between individual and organisational attitudes toward coercion—showed no meaningful association with moral distress.

### Qualitative results

**Fig. 1:**
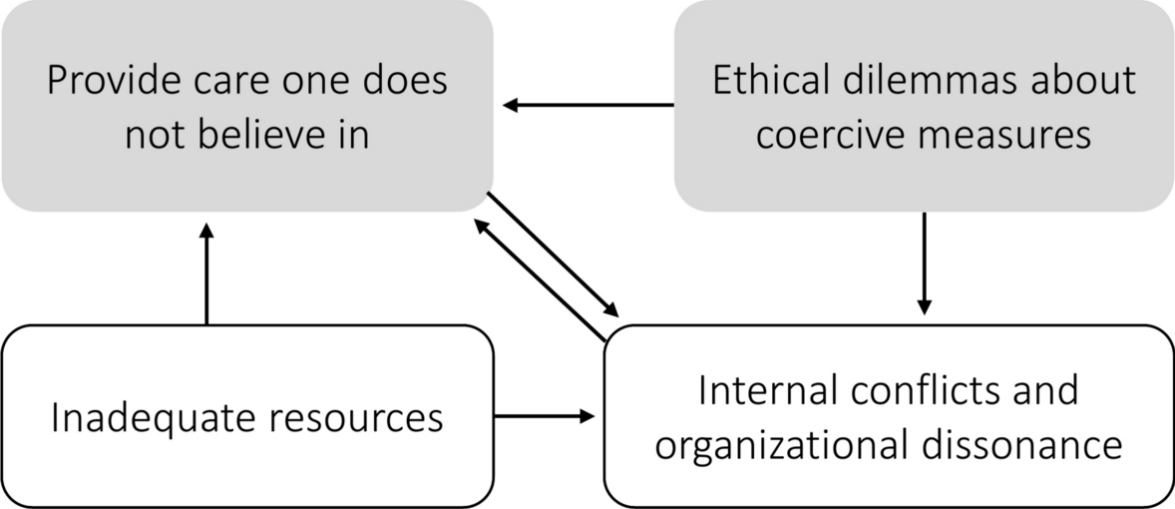
Themes (grey-shaded) and subthemes influencing moral distress in inpatient child and adolescent psychiatric staff

We created themes to capture the core experiences shared by participants: “Provide care one does not believe in”, with the subthemes “Inadequate resources” and “Internal conflicts and organisational dissonance”; and the theme “Ethical dilemmas about coercive measures”. See Fig. 1 for a schematic depiction of the themes.

#### Theme: Providing care one does not believe in

Participants frequently reported being compelled to provide care that conflicted with their beliefs, ethics, or professional standards, resulting in moral distress. A key source of this distress was witnessing patients receive inadequate or no treatment, as many participants described having to prioritise acute situations, leaving constructive and effective care deprioritised.

Margareta, nurse: [On sources of moral distress] Not working according to known treatment findings that shorten treatment times and provide substantially higher quality, and thus better for everyone in every conceivable way.

#### Subtheme: Inadequate resources

One aspect of *Providing care one does not believe in* was the perception of inadequate resources, which affected the quality of care and contributed to feelings of moral distress. Specifically, participants described a lack of available hospital beds, and a harmful ward environment, affecting patient care and recovery negatively. Also, a high workload without adequate recovery time was seen as exacerbating staff burnout and diminishing staff ability to offer optimal patient care. In addition, a lack of time to sufficiently attend to patients was described as leading to feelings of inadequacy and moral distress.

Lars, nurse: [I experience moral distress] when I feel that a patient needs my time in a way that I cannot give, because many others need it at the same time.

Many participants stressed the value of experienced staff in enhancing care quality, ensuring patient safety, and reducing coercive measures. However, this expertise was not perceived to be sufficiently recognised or retained by the organisation. Further, participants described that frequent staff turnover and shortages of nurses and psychiatrists disrupted care continuity. To compensate for this shortage, participants reported that working overtime had become commonplace.

Anna, physician: Time and experience are so important. When the organisation is under pressure, experienced staff do not stay, the risks and pressure on us who dare to stay increase. [It is important to actually provide] reasonable conditions for experienced staff to stay—and new ones to dare to stay until they become experienced.

Staff also described a sharp increase in patients with eating disorders as a growing contributor to moral distress, reporting feelings of unpreparedness and substantial resource gaps.

Linnea, social worker: The recent wave of patients with eating disorders has shown major resource problems to meet these patients in an ethically correct and treatment-effective way.

#### Subtheme: Internal conflicts and organisational dissonance

Another aspect of *Providing care one does not believe in* was experiences of internal conflicts and organisational dissonance leading to moral distress. The subtheme captures the problems encountered due to a lack of consensus within teams, differences in treatment approaches, and structural deficiencies in collaboration with internal and external care providers.

In detail, participants frequently pointed out disagreements among team members (commonly with physicians), concerning the best care strategies for patients, leading to emotional friction and dissatisfaction. A major influence on moral distress was a sense of not being able to voice disagreements or concerns about decisions perceived as detrimental to patients.

Nils, psychiatric aide: [On sources of moral distress] Conflicts with physicians. Difficult to cooperate regarding the patient’s needs.

Additionally, poor collaboration with external care providers, such as social services or outpatient care units, resulted in prolonged patient stays and re-admissions.

Olof, physician: [It’s morally distressing] when patients and families do not get what they need and are entitled to from other care providers that I have no control over, such as outpatient care and social services.

There was a shared perception that patients with eating disorders faced coercive measures more frequently than other patient groups. The nature of these measures, such as nasogastric tube feeding, were a source of disagreement and conflict among staff.

#### Theme: Ethical dilemmas about coercive measures

Coercive measures could invoke strong emotional and ethical reactions among staff members. Some grappled with feeling like “the bad guy” which created moral dissonance, particularly when working with patients with an eating disorder. As mentioned above, the use of nasogastric tube feeding, was seen as very ethically challenging and controversial.

Marianne, nurse: The first time I tube-fed a restrained patient, I had a surreal feeling of being in a horror movie set in a mental hospital, and *I* was one of the bad guys.

Some participants described the work with coercive measures as particularly morally distressing. These participants experienced strong negative emotions and found working with coercive measures difficult. They described how it had a negative impact on their private life in forms of stress symptoms, difficulty sleeping, decreased appetite, ruminations and doubts about one’s own morals.

Fredrik, nurse: [The work of coercive measures results in] that you, sometimes, feel hopelessness and lose commitment. That you sometimes take work home with you and dwell on traumatic coercive measures in an unhealthy way that leads to poorer recovery [for yourself].

Some described that using coercive measures became particularly morally distressing when they clearly saw patient and relatives suffer from it. Descriptions ranged from seeing patients in pain, noticing that the patient did not comprehend the aim of the coercive measure, to having parents question the coercive measure.

Sofia, nurse: [How I am affected] depends on how the patient handles the coercive action as such. If it is noticeably difficult for the patient, I can bring it home with me and experience an uneasy feeling and sadness about what has happened.

Further, situations when coercive measures had to be used but could have been avoided were seen as especially morally distressing. For example: coercive measures that were triggered by staff; that took place routinely; did not lead forward in treatment; were consequences of lack of resources; were legally questionable; or that staff did not agree with. This also included situations where coercive measures lead to negative treatment effects, such as spoiling the alliance with the patient or reinforcing self-harm.

Anders, psychiatric aide: [It’s morally distressing] when there are coercive measures because the staff has failed to listen to the patient and when the staff “triggers” a situation that could have been avoided. Unfortunately, this happens quite often.

In contrast, many viewed the use of coercive measures as an integral part of their work and felt neutral towards them. This neutrality often stemmed from a belief in the ethical appropriateness of the measures when truly needed. Both participants who felt emotionally unaffected and participants who were morally distressed by the work with coercive measures, saw that coercive measures could be necessary, especially in acute or dire situations.

Lars, nurse: It is difficult. But it is also necessary when a coercive measure is decided. The options are simply worse. The fact that the work is difficult is not the same as bad or wrong. The work becomes difficult because it involves empathic ability and a willingness to help the patient to the better alternative.

### Triangulation of quantitative and qualitative data

The joint display integration of quantitative and qualitative data revealed converging findings (Table 3). Younger and less experienced professionals reported higher levels of moral distress (quantitative data, Table 1) also evident in the qualitative data, where participants described how greater professional experience contributed to lower levels of moral distress and improved care quality. Quantitatively, physicians reported the highest levels of moral distress. While less explicitly outlined in the qualitative data, interprofessional conflicts between other staff and physicians regarding treatment decisions and team dynamics were described as a source of moral distress. The strong association between low social support and moral distress was less evident in qualitative data, although team conflicts and a perceived inability to voice concerns were described. Low job control was not particularly emphasised in either dataset.

**Table 3:** Integration of quantitative and qualitative results on moral distress in child and adolescent psychiatry staff

Key factors	Quantitative results	Qualitative results
Age and experience	Moral distress decreased moderately with age and with over five years of inpatient CAP experience	Experienced staff reported lower moral distress and helped mitigate team distress. Inexperienced staff contributed to higher moral distress
Occupation	Physicians reported highest moral distress compared to psychiatric aides and nurses	Frustration with physicians about decision conflicts and team dynamics
Resources and work environment	High job demands and low social support were strongly associated with moral distress	High workloads and insufficient resources linked to moral distress and lower care quality
Coercive measures	Positive perception of coercion moderately associated with lower moral distress Weak positive correlation between coercion frequency in ward and moral distress	Ethical dilemmas surrounding coercion, particularly with eating disorder patients, amplified moral distress
Job retention	Intention to resign from job was strongly associated with higher moral distress	Emotional toll led to burnout and intention to resign

Both our data sources identified resource constraints as a key contributor to moral distress. In the qualitative analysis, participants described high workloads, insufficient recovery time, and limited hospital beds as barriers to providing effective care. That was in line with the strong quantitative association between high job demands and moral distress.

The negative impact of moral distress on job satisfaction and workforce retention was strongly suggested in both datasets. Quantitative data found a strong association between moral distress and intention to resign, while the qualitative analysis provided deeper insight into the emotional burden and long-term consequences of moral distress, including burnout and compromised recovery outside of the work setting.

Ethical challenges related to coercive measures were another important source of moral distress. Qualitative data captured the ethical and emotional difficulties associated with these practices, particularly in the care of patients with eating disorders. These challenges were less clear quantitatively: a weak positive correlation was observed between moral distress and reported frequency of coercive measures. However, staff who viewed coercive measures as ethically acceptable reported less moral distress.

## Discussion

We found high levels of moral distress among healthcare professionals in inpatient CAP, with higher moral distress related to inexperienced staff, a demanding work environment, and ethical conflicts about coercive measures. Inadequate resources and high staff turnover emerged as consistent and substantial systemic or organisational problems. Younger, less experienced professionals and physicians appeared particularly vulnerable to such distress, possibly because of limited coping strategies, heavier responsibility, and increased exposure to ethically challenging situations. These support an interplay of individual, professional, and systemic factors in shaping moral distress, thus emphasising the need for targeted interventions to address resource constraints, enhance support for inexperienced staff, and create ethically supportive work environments.

An important finding is the higher level of moral distress among staff in CAP inpatient care compared to those in other healthcare sectors. With the exception of one study with Turkish nurses [25], previous studies using the SCQ reported lower levels of moral distress among healthcare professionals [22, 34, 35], including staff in Swedish adult psychiatric inpatient care [36]. Moreover, an interview study with nurses and nurse assistants in Swedish CAP found that constrained nursing practices and a lack of clear leadership intensified the ethical tensions experienced by staff [37]. This implies that working with mentally ill youth and their families in inpatient care might present unique ethical challenges.

The inverse relationship between moral distress, age, and professional experience corresponds to previous research [25], suggesting that experience enhances coping mechanisms and ethical decision-making [38]. Experienced professionals may find it easier to reconcile personal values with organisational constraints and engage effectively in interprofessional dialogues, thus fostering resilience in ethical dilemmas. Qualitative findings corroborate this, reporting that moral distress often arises from internal conflicts and systemic pressures, which may be harder for less experienced staff to navigate [16].

However, in addition to better support to early-career professionals, alternative explanations to this association merit further investigation. Generational differences may play a role, as younger professionals may have different expectations of their work environment and a stronger emphasis on personal values in clinical practice, which could heighten moral distress. Changes in training curricula could also contribute, as modern education increasingly emphasises ethical reflection, patient autonomy, and trauma-informed care. While this fosters awareness of ethical dilemmas, it may not fully equip staff with the practical strategies needed to navigate these challenges within the constraints of inpatient psychiatric care, potentially increasing frustration and distress. Lastly, self-selection effects may explain why moral distress appears lower among experienced staff—over time, those who struggle with high moral distress may leave inpatient care, while those who remain may have been less prone to experiencing it from the outset or developed greater resilience.

Our results show that contrary to much research centred on nurses [8, 39], physicians reported the highest moral distress levels, while psychiatric aides reported the lowest levels. This is surprising, given that physicians typically possess more control within their work environment—a factor often linked to reduced stress. However, a qualitative study on moral distress in psychiatrists described their experience of balancing difficult ethical dilemmas while feeling great moral responsibility [40]. Thus, physicians’ heightened moral distress can reflect the impact of responsibilities inherent in their role, including decision-making in complex ethical situations. Hierarchical organisational structures may also exacerbate this distress by isolating decision-making and hindering cross-professional consensus, as reported in the subtheme “Internal conflicts and organisational dissonance”.

A possible harmful work environment impact on staff is suggested in both our qualitative and quantitative findings. Consistent with previous research [8, 41], key contributors to moral distress were high job demands, inadequate resources, and insufficient social support at work. Moreover, our results align with prior studies describing the importance of leadership support, organisational clarity, and the resolution of team disagreements in mitigating these challenges [42]. To address moral distress through improvements of the work environment, efforts should prioritise resource allocation to alleviate job demands and initiatives to strengthen team dynamics, thereby enhancing social support and overall workplace cohesion.

Moral distress was strongly associated with considering resigning, aligning with previous research that associates moral distress with job dissatisfaction and a propensity to quit [3, 5]. A high staff turnover in healthcare, particularly pronounced among young and inexperienced nurses, underscores this trend [43, 44]. Our qualitative results suggest that inexperienced staff not only faced higher moral distress, but also contributed to the moral distress of their more seasoned colleagues, who as a result may bear additional responsibilities. This indicates a problematic cycle: a high turnover of experienced staff exacerbates the stress for remaining personnel and compromises the work environment, potentially prompting further departures and perpetuating the cycle.

The inverse relationship between positive attitudes toward coercive measures and moral distress reveals a nuanced ethical dynamic. Contrary to previous findings from inpatient CAP [13]—suggesting that ethical dilemmas about coercive measures are rare due to their perceived necessity—our study reveals a more complex relationship. Consistent with research in adult psychiatry [45], our participants recognised coercive measures as an occupational necessity; however, they described these measures as morally distressing when perceived as preventable or unwarranted. Further, our findings suggest more frequent coercive measure use contributes to moral distress, but this association may be bidirectional: increased moral distress or resource shortages could cause an increased reliance on coercive practices. Interestingly, the growing pressure to minimise coercion has itself been linked to moral distress, in turn stemming from the tension between expectations to reduce coercive practices and the practical necessity of ensuring safety for patients and staff when alternatives are unviable [46]. Consequently, the impact of coercive measures on moral distress may vary with individual attitudes and specific circumstances.

Staff specifically reported moral distress related to treating patients with eating disorders, due to lacking resources and expertise for effectively treating these patients. Previous research on patients with anorexia nervosa has linked moral distress to conflicts between the principles of beneficence, non-maleficence, and respect for autonomy [47]. Our findings align with global reports of an alarming rise in the number of patients with eating disorders in CAP inpatient care [48]. These trends underscore the need for an urgent investigation into the unique ethical and systemic challenges of treating children and adolescents with eating disorders.

### Strengths and limitations

This study has several notable strengths. As the first study to quantitatively examine moral distress in CAP inpatient care staff, it includes extensive geographical coverage and a substantial sample size. The use of validated instruments contributes to reliability and generalizability, while the mixed-methods approach integrates qualitative and quantitative insights, enriching the depth of analysis. Further, the qualitative data provides a nuanced understanding of the CAP inpatient work environment and increases transferability.

However, certain limitations warrant discussion. The cross-sectional design limits causal inferences, allowing only observations of associations. Further, as with all survey research, our sampling might have introduced selection bias, as engaged or informed participants could be overrepresented. This could affect the generalisability of the findings, as those with strong opinions or personal experiences related to moral distress may have been more likely to participate. Nevertheless, the respondents represented 14 of Sweden’s 18 CAP inpatient care regions, thus reflecting substantial diversity. No units refused participation, and an internal missing data analysis found no systematic differences between respondents and non-respondents.

The dimensionality of the SCQ remains debated; though designed to measure internal and external stressors recent evidence supports its one-dimensionality [24]. While this approach aligns with current validation, total scores may obscure individual differences in contributing factors. Also, overlapping constructs—such as job demands and the frequency of ethical dilemmas—warrant careful interpretation in correlational analyses.

Perceived frequency of coercive measure use is a subjective measure and reflects the respondents’ perceptions; hence, it could have been affected by the frequency of recent coercive measure use when completing the questionnaire. However, such perceptions remain valuable for exploring associations with the use of coercive measures as they capture respondents interpretation of their work environment.

By explicitly asking about coercive measures, we may have influenced participant responses regarding moral distress. While coercive practices are a likely source of moral distress in psychiatry, our survey design might inadvertently have directed participants’ focus toward these experiences, potentially overlooking other contributing factors. This could have resulted in an overemphasis on coercion-related distress, compared to other morally challenging situations. Future research in this context might consider broader, open-ended approaches to capture a comprehensive range of factors contributing to moral distress.

Our findings align with research from adult psychiatric inpatient care, indicating that the systemic and ethical challenges driving moral distress may be quite consistent across psychiatric inpatient settings. This suggests that the dynamics of inpatient psychiatric care, rather than patient age, play a vital role in shaping moral distress. While cultural and systemic differences may influence specific outcomes, the broader relevance of these findings might extend to similar psychiatric contexts internationally.

Finally, for trustworthiness [49], we aimed to ensure credibility through investigator triangulation, dependability by following Braun and Clarke’s analytical standards, and confirmability with an audit trail. While our insider knowledge allowed for a nuanced interpretation of the data, it may also have shaped our perspectives during analysis. To address this, we engaged in rigorous reflexivity throughout the process. Resource constraints meant we could not include an external review, but we believe working collaboratively helped ensure a more balanced and credible analysis.

### Future directions

To address the high levels of moral distress and staff turnover in CAP inpatient care, our findings support the need to reduce job demands through resource augmentation, workload reduction, and improved staffing ratios. Strategies such as increasing the proportion of educated staff, providing targeted education, and decreasing patient-to-staff ratios should be prioritised. Further, given the level of moral distress reported among physicians, future initiatives might consider implementing occupation-specific support strategies tailored to their unique professional challenges. Breaking the cycle of staff turnover is vital to preserve and build the knowledge base grounded in experience. Long-term retention strategies are essential to sustain a skilled and experienced workforce capable of meeting the complex demands of CAP inpatient care.

## Conclusions

Moral distress among CAP inpatient care staff across Sweden is pronounced, particularly among younger, less experienced professionals, and physicians. It is associated with workforce attrition, systemic resource inadequacies, and the ethical challenges of coercive measures. Targeted interventions, further research, and improved resource allocation are essential to mitigate this occupational hazard and ensure sustainable healthcare provision for the most mentally disordered children and adolescents.

## Supplementary Information

Supplementary Material 1: See Appendix for additional descriptions of methods, additional detailed results, and visualisations of data distribution. Further, we provide an English translation of the questionnaire.

## Data Availability

The datasets generated and analysed during the current study are not publicly available due to ethical restrictions. However, they can be made available upon reasonable request to the corresponding author, in accordance with data protection regulations and ethical guidelines.

## Abbreviations

CAP: Child and adolescent psychiatry
SACS: Staff Attitude to Coercion Scale
SCQ: Stress of Consciousness Scale
DSCQ: Demand Support Control Questionnaire
