# Supplementary material for: Moral distress among inpatient child and adolescent psychiatry staff: a mixed-methods study of experiences and associated factors
Source: Child Adolesc Psychiatry Ment Health. 2025 Feb 28;19:16. doi: 10.1186/s13034-025-00868-7 (PMC11871634; doi:10.1186/s13034-025-00868-7)
Supplement: Supplementary file 1 — Supplementary Material 1: See Appendix for additional descriptions of methods, additional detailed results, and visualisations of data distribution. Further, we provide an English translation of the questionnaire. [file 13034_2025_868_MOESM1_ESM.pdf]

Appendix to
Moral Distress Among Inpatient Child and Adolescent Psychiatry Staff: A Mixed-Methods Study of Experiences and Associated Factors

Table of contents

Additional details on the qualitative method 2
Analysis tree 2
Additional details on the quantitative method 2
SCQ (Stress of Conscience Questionnaire) 2
SACS (Staff Attitude to Coercion Scale) 2
DSCQ (Demand Support Control Questionnaire) 3
Additional data 3
Analysis of missing data 3
SCQ subscales: Internal and external distress 3
Age groups and Total SCQ 3
Perceived frequency of coercive measure use 4
Professional experience and Total SCQ 4
Occupational categories and Total SCQ 4
Demand Support Control Questionnaire (DSCQ) 5
Staff Attitude to Coercion Scale (SACS) 5
Plots examining score distributions 7
References 15
Questionnaire 16
Moral Distress Among Staff in Inpatient Child and Adolescent Psychiatry in Sweden 16
Demographic information 17
[The Stress of Conscience Questionnaire (SCQ)] 18
[The Staff Attitude to Coercion Scale (SACS)]: individual rating 18
[The Staff Attitude to Coercion Scale (SACS)]: organisational rating 18
[Demand Support Control Questionnaire (DSCQ)] 19
Intention to leave job 19
Free text questions 19

Additional details on the qualitative method

Word counts for individual questions were as follows: "What in your work leads to moral distress?" had 1808 words, "How does the work with coercive measures affect you?" contained 1378 words, and "Is there anything else you want to add?" resulted in 981 words. Answers ranged from a single word to a maximum of 228 words but typically spanned two to three sentences.

Analysis tree

The construction of the subtheme *Inadequate resources* is here used as an illustration of the reflexive thematic analysis following Braun & Clarke (1,2). The participant's answer:

> "Difficult work environment due to workload - one never has time to finish the work."

was coded as "lack of time" and "workload". During phase three (searching for themes) we saw a clear pattern where many codes concerned lacking resources and chose *Inadequate resources* as a candidate theme. When we defined and reviewed the theme in phase four, we started with four subthemes: *Time, Staff, Hospital bed capacity,* and *Experience and competence*. The subthemes were, however, revised in the following phases, after realizing that they appeared to be different ways of describing the same problem. For example, a staff shortage will most likely generate time pressure, and an overcrowded ward will result in insufficient time and staff to maintain the same quality of care. We decided to use *Inadequate resources* as a subtheme to *Providing care one does not believe in* since *Inadequate resources* clearly affected the ability to provide care and, thus distress being the effect of the overarching theme more than the subtheme.

Additional details on the quantitative method

We considered a Cronbach's α of 0.7–0.95 acceptable (3). Cronbach's α is a measure of internal consistency, reflecting the degree to which items within a scale are correlated and measure the same underlying construct.

SCQ (Stress of Conscience Questionnaire)

For this study, the SCQ demonstrated good internal consistency with a Cronbach's α of 0.84.

SACS (Staff Attitude to Coercion Scale)

The SACS had not previously been translated into Swedish, so we secured developer professor Tonje Lossius Husum's permission to translate and employ it in our research. The initial translation underwent review by a bilingual Norwegian-Swedish nurse, with subsequent revisions informed by her feedback. Higher scores indicate a more positive attitude towards coercive measures. Hence, scores for items 3-4,8,13-15 (subscale Coercion as offending) were inverted when calculating total mean scores per item.

For this study, the total SACS score demonstrated good reliability concerning internal consistency, with Cronbach's α of 0.81 for individual attitudes and 0.78 for perceived organizational attitudes. Analyses of subscales across individual and workplace perspectives generally reflected good to moderate internal consistency; *Coercion as Offending* (α = 0.70 vs. 0.66), *Coercion as Care and Security* (α = 0.72 vs. 0.74), and *Coercion as Treatment* (α = 0.76 vs. 0.70).

DSCQ (Demand Support Control Questionnaire)

For this study, the DCSQ demonstrated reliable Cronbach's α values: (job demands), 0.63 (job control), and 0.83 (social support), 0.79 indicating moderate to strong reliability.

Additional data

Analysis of missing data

Table A1. Missing data analysis in study of moral distress in staff working in inpatient child and adolescent psychiatric care in Sweden

Variable	Mean SCQ (no missing)	Mean SCQ (with missing)	95% CI	Results (t, df, p-value)
Age	42.10	37.81	-1.34–9.91	t = 1.54, df = 39.10, p = 0.13[a]
Gender	N/A	N/A	N/A	p = 0.48[b]
Work experience	N/A	N/A	N/A	p = 0.59[b]
Occupation	N/A	N/A	N/A	p = 0.60[b]
Region of work	N/A	N/A	N/A	p = 0.47[b]
SCQ	62.86	49.08	-8.05–35.61	t = 1.34, df = 16.16, p = 0.20[a]

Notes: N/A = Not applicable. [a] Welch two-sample t-test, [b] Fisher's Exact Test for count data.

SCQ subscales: Internal and external distress

Table A2. Internal and external demand SCQ subscales scores in staff working in inpatient child and adolescent psychiatric care in Sweden, N=106

SCQ subscale	Median (IQR)	Mean (SD)
Internal demands	30.5 (17.0, 45.0)	32.2 (19.6)
External demands	27 (14.0, 42.8)	29.6 (20.5)

Age groups and Total SCQ

Table A3. Total SCQ score by age group among staff working in inpatient child and adolescent psychiatric care in Sweden, N=106

Age group (yrs)	N (%)	Total SCQ Median (IQR)
21–30	22 (21%)	66.0 (36.3, 95.0)

31–40	31 (29%)	78.5 (48.5, 99.0)
41–50	24 (23%)	60.0 (34.0, 85.0)
51–60	25 (24%)	46.0 (25.0, 57.0)
61+	4 (4%)	26.0 (17.0, 31.0)

Perceived frequency of coercive measure use

Table A4. Perceived frequency of coercive measure use at their unit by staff within inpatient child and adolescent psychiatric care in Sweden, N=91

Frequency coercive measure use	N (%)
Every day, *n (%)*	2 (2.2)
Often (at least once a week), *n (%)*	17 (18.7)
Sometimes (a few times a month), *n (%)*	33 (36.3)
Rarely (a few times a year), *n (%)*	25 (27.5)
Very rarely (once a year), *n (%)*	14 (15.4)
Never, *n (%)*	0 (0.0)

Professional experience and Total SCQ

Table A5. Total SCQ scores among staff by professional experience of inpatient child and adolescent psychiatric care

Work experience	N (%)	Total SCQ Median (IQR)
Less than a year	12 (11%)	64.5 (49.75, 92)
1-2 years	36 (34%)	59.5 (39.0, 88.3)
3-5 years	22 (21%)	74.0 (61.5, 96.8)
6-8 years	9 (9%)	47.0 (30.0, 68.0)
8-10 years	7 (7%)	45.0 (8.5, 69.0)
More than 10 years	20 (19%)	37.0 (20.3, 65.8)

Occupational categories and Total SCQ

Table A6. Total SCQ scores by occupational categories among staff working in inpatient child and adolescent psychiatric care in Sweden, N=106

Comparison	Hodges-Lehman estimator	95% CI	p-value
Physician vs. Social worker or treatment provider	-10	-48, 24	1

Physician vs. psychiatric aide	35	17, 52	0.003
Physician vs. Nurse	27	6, 47	0.058
Social worker or treatment provider vs. Psychiatric aide	22	-9, 52	0.670
Social worker or treatment provider vs. Nurse	13.5	-19, 49	1
Psychiatric aide vs. Nurse	9	-10, 25	1

Notes: P-values adjusted using the Holm method to account for multiple comparisons.

Demand Support Control Questionnaire (DSCQ)

Table A7. Demand support control questionnaire (DSCQ) scores among staff working in inpatient child and adolescent psychiatric care in Sweden, N=90

Subscales[a]	Mean	SD
Job demand	13.97	2.97
Job control	19.16	2.57
Social support	18.24	3.47

Notes: [a] Higher scores indicate higher perceived job demands/job control/social support.

Staff Attitude to Coercion Scale (SACS)

Table A8. Staff Attitude to Coercion Scale (SACS) among staff working in inpatient child and adolescent psychiatric care in Sweden, N=90

	Median	Mean	SD
**Total mean score per item[a]**			
Individual	3.20	3.16	0.58
Workplace	3.20	3.21	0.50
Subscales			
**Coercion as offending**			
Individual	2.83	2.83	0.75
Workplace	2.83	2.89	0.63
**Coercion as care and security**			
Individual	3.83	3.88	0.64
Workplace	3.83	3.83	0.62
**Coercion as treatment**			
Individual	2.33	2.38	0.91

Workplace	2.67	2.64	0.83

Notes: [a] Higher scores indicates positive attitudes to coercive measures. Scores were inverted for subscale *Coercion as offending*.

Plots examining score distributions

Moral distress and gender

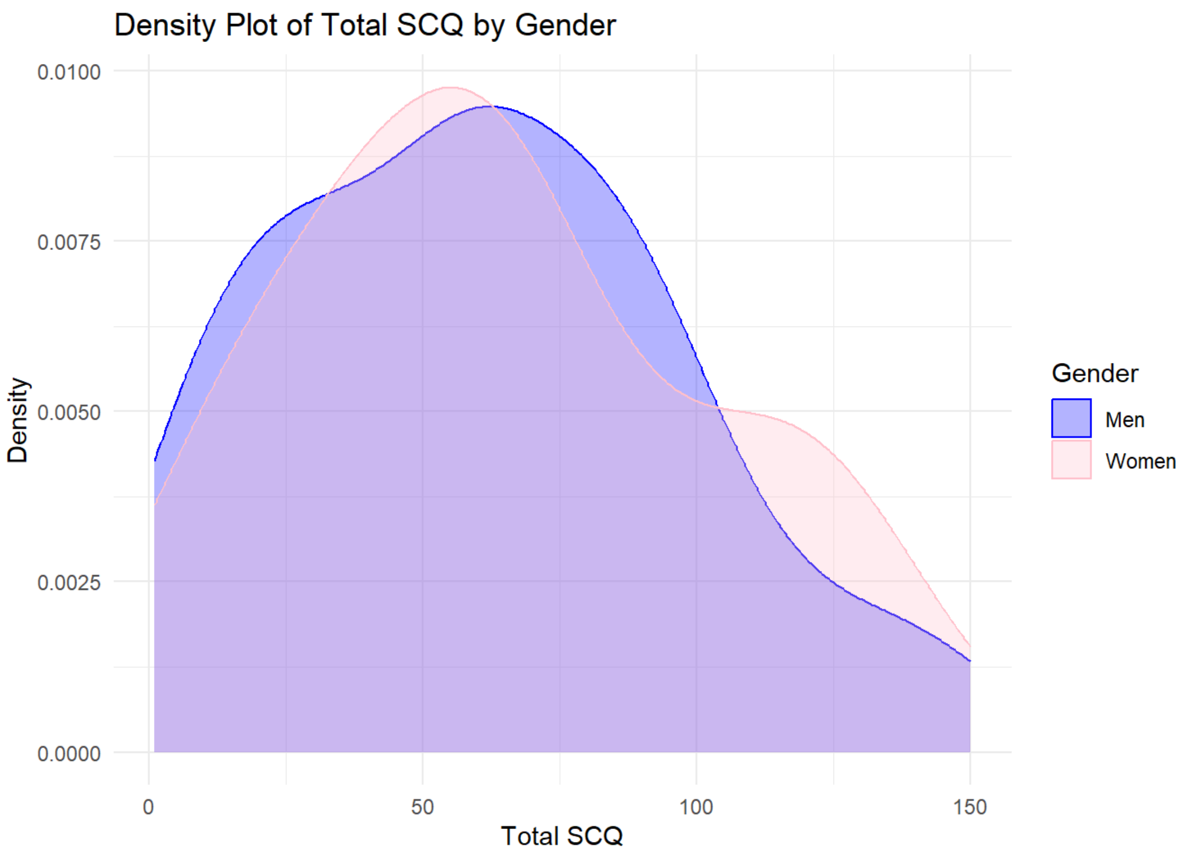

Moral distress and age

Grey shading represents the 95% confidence interval around the red trend line.

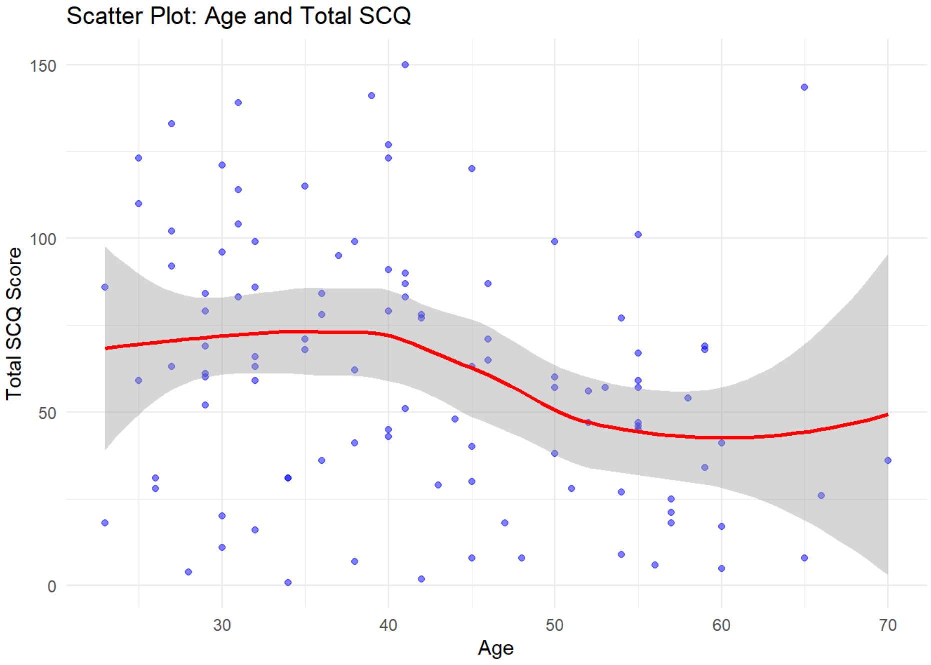

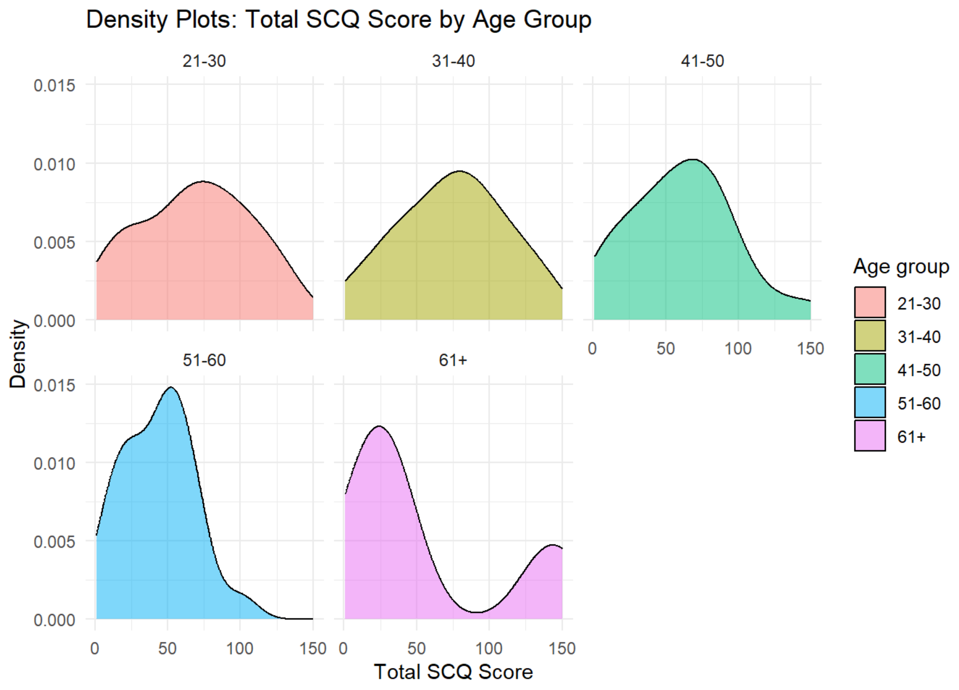

Moral distress and years of professional experience

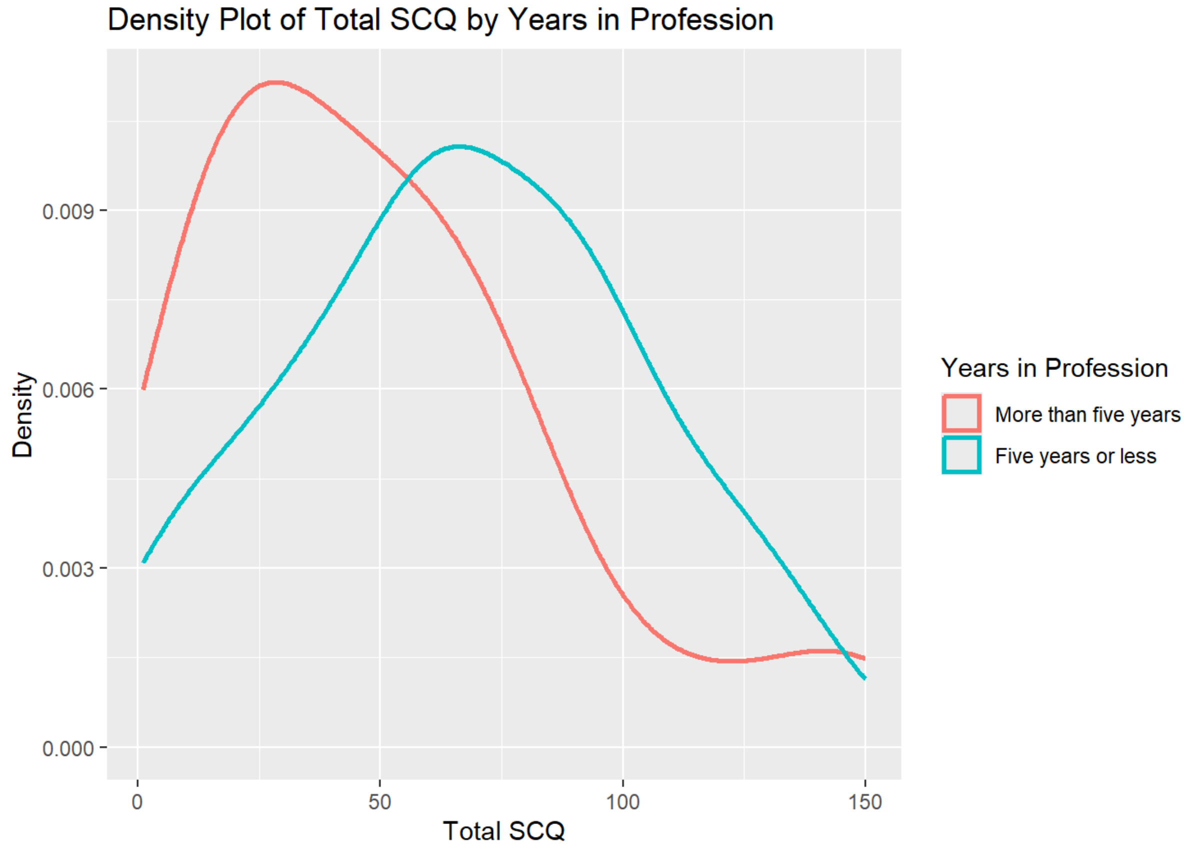

Moral distress and occupation

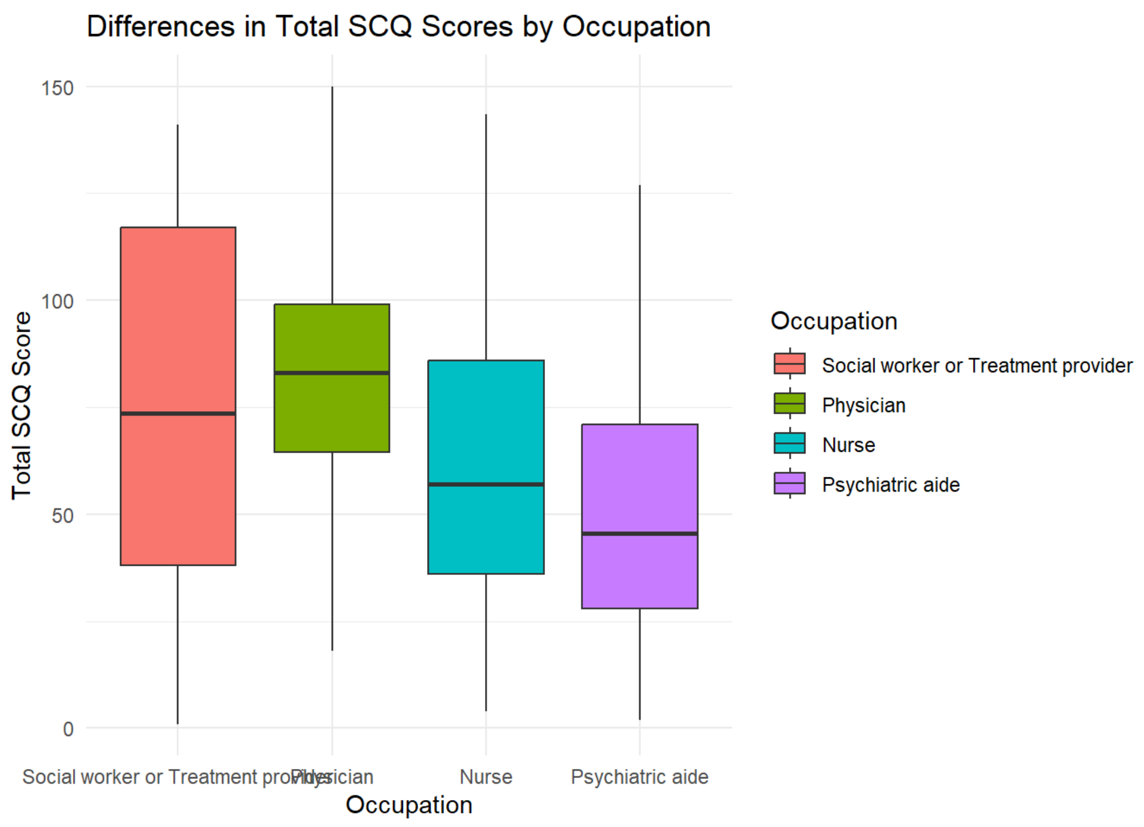

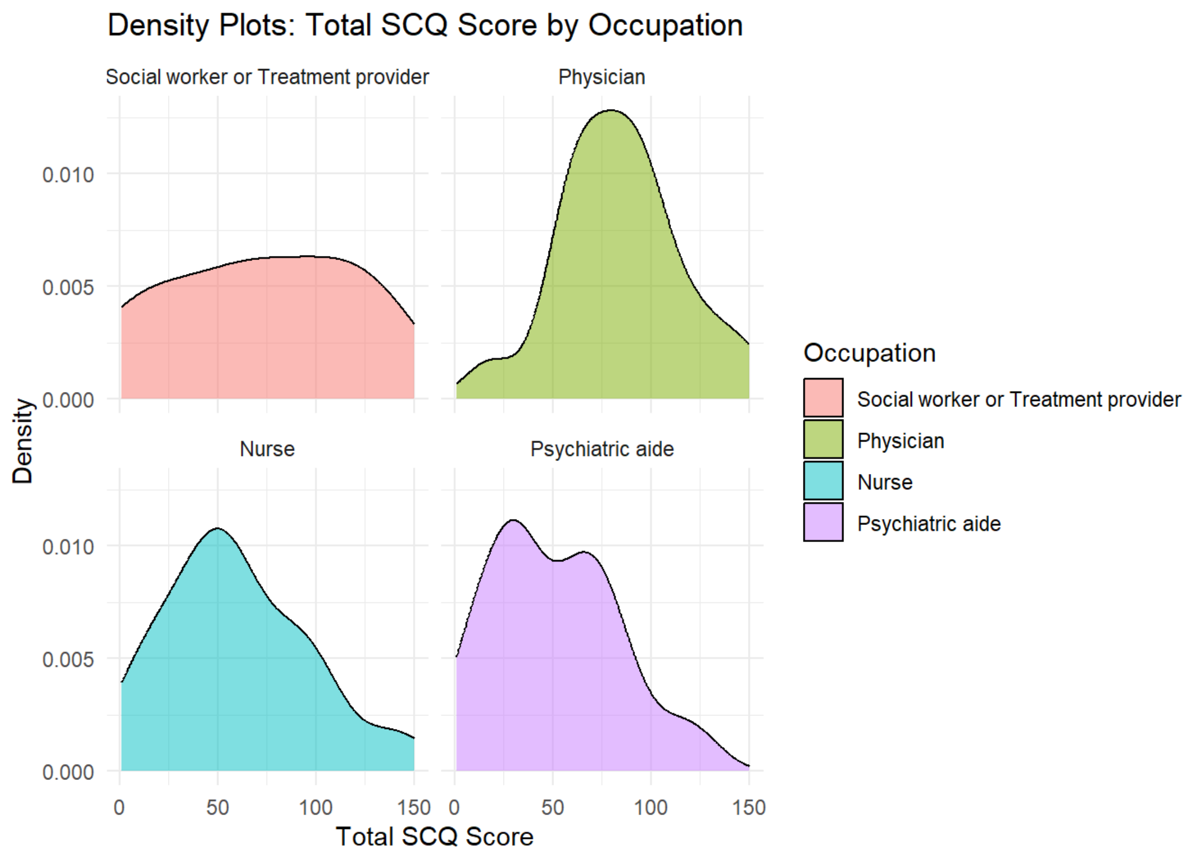

Moral distress and intention to resign from job

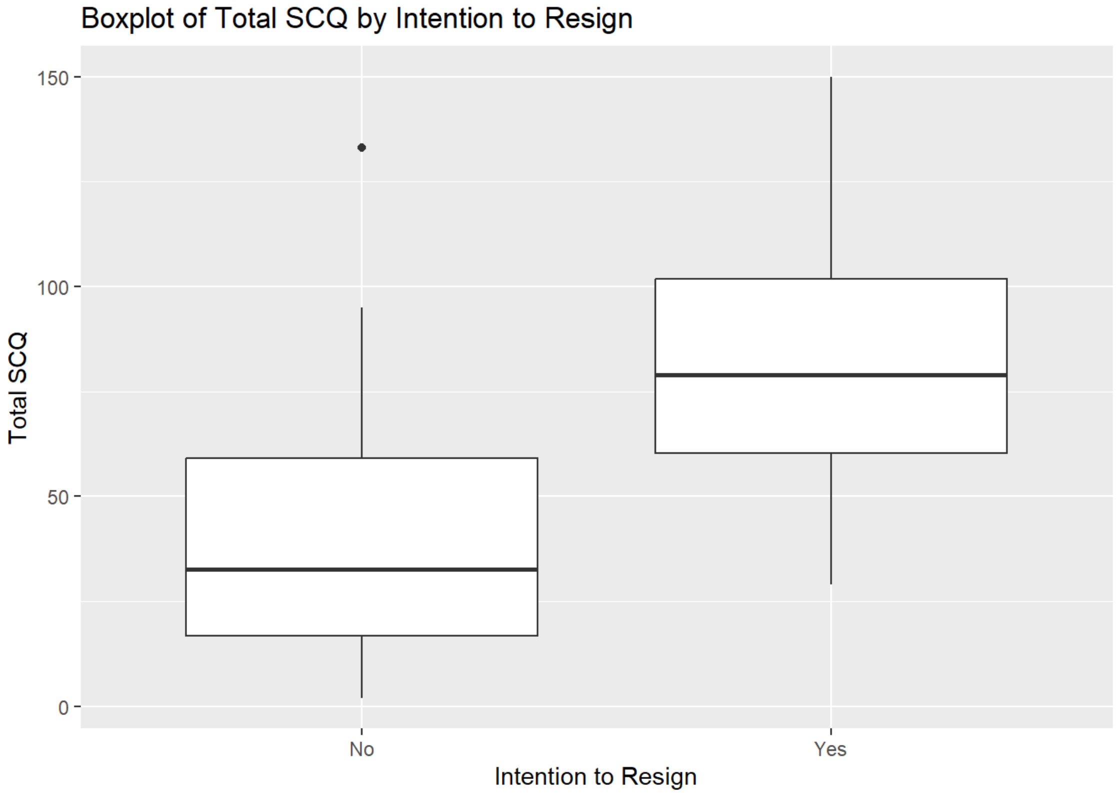

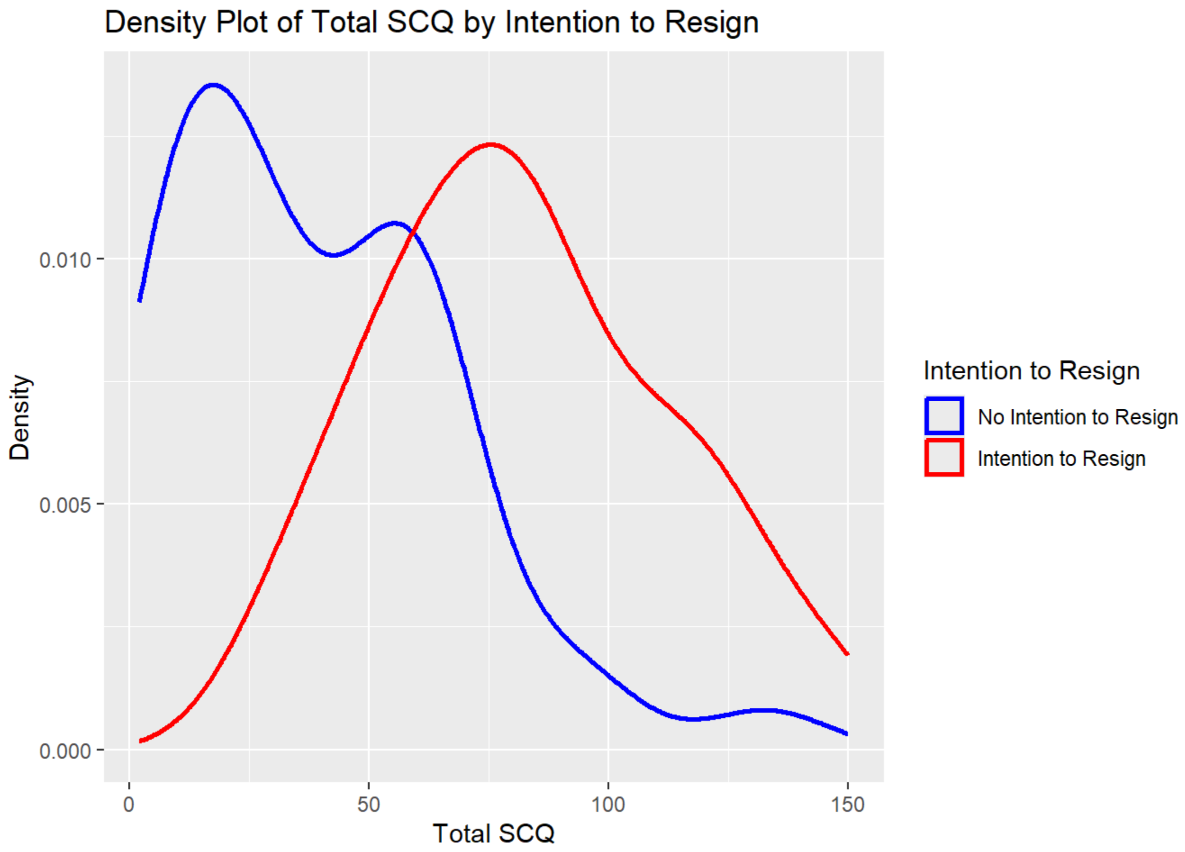

Moral distress and work environment

Job demand (DSCQ)

Grey shading represents the 95% confidence interval around the red trend line.

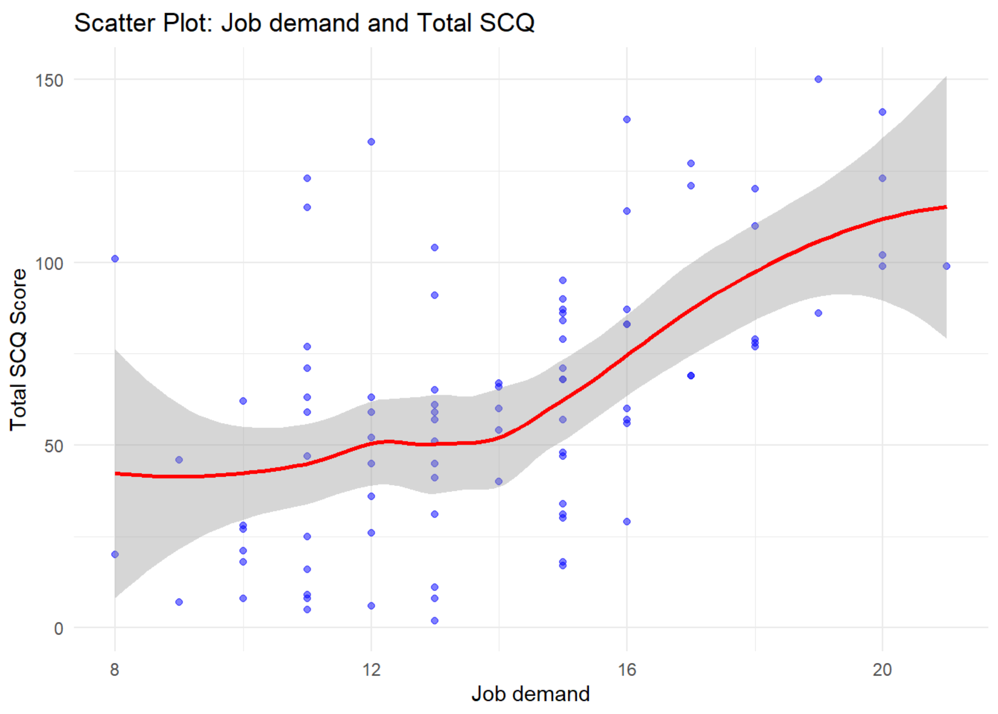

Job control (DSCQ)

Grey shading represents the 95% confidence interval around the red trend line.

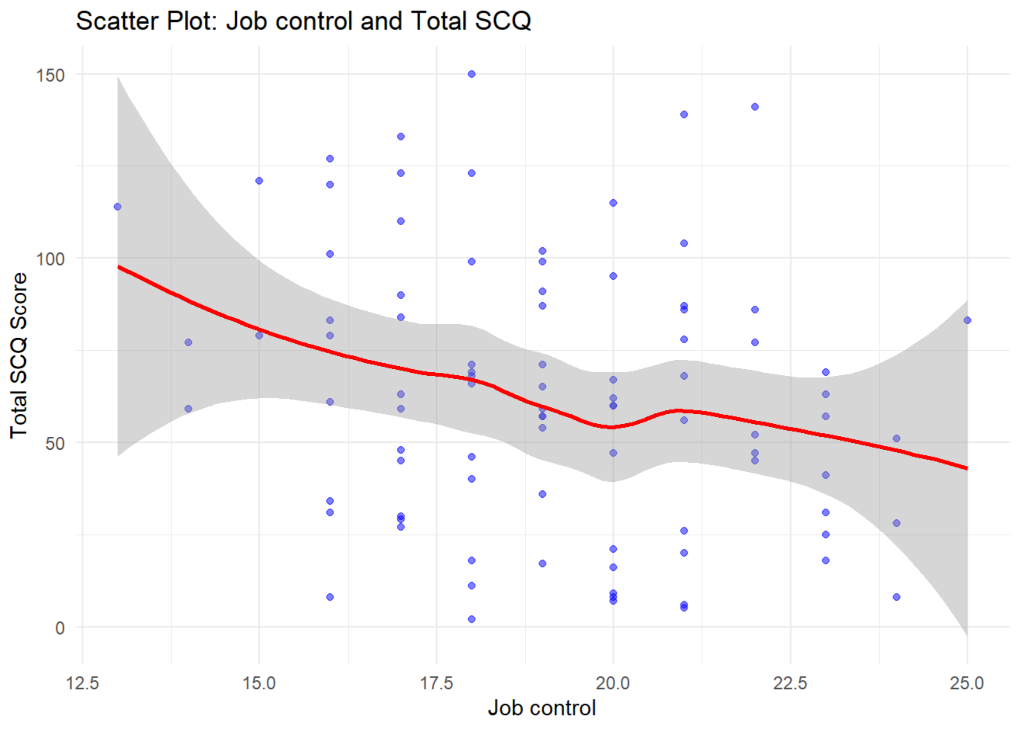

Social support (DSCQ)

Grey shading represents the 95% confidence interval around the red trend line.

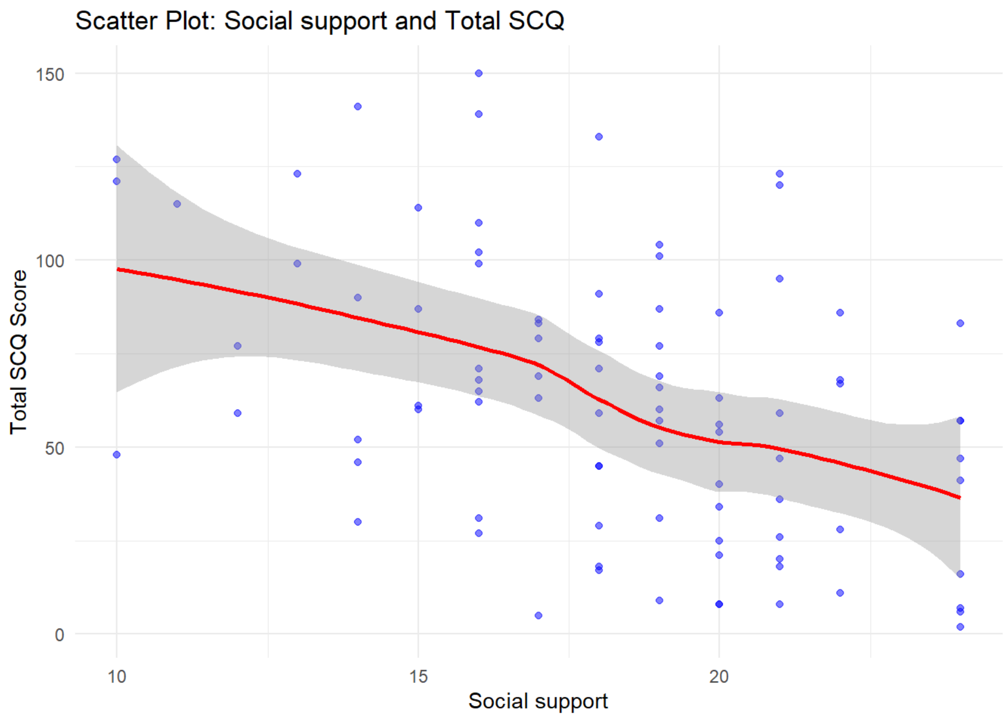

Moral distress and Staff Attitude to Coercion Scale (SACS)

Total score SACS

Grey shading represents the 95% confidence interval around the red trend line.

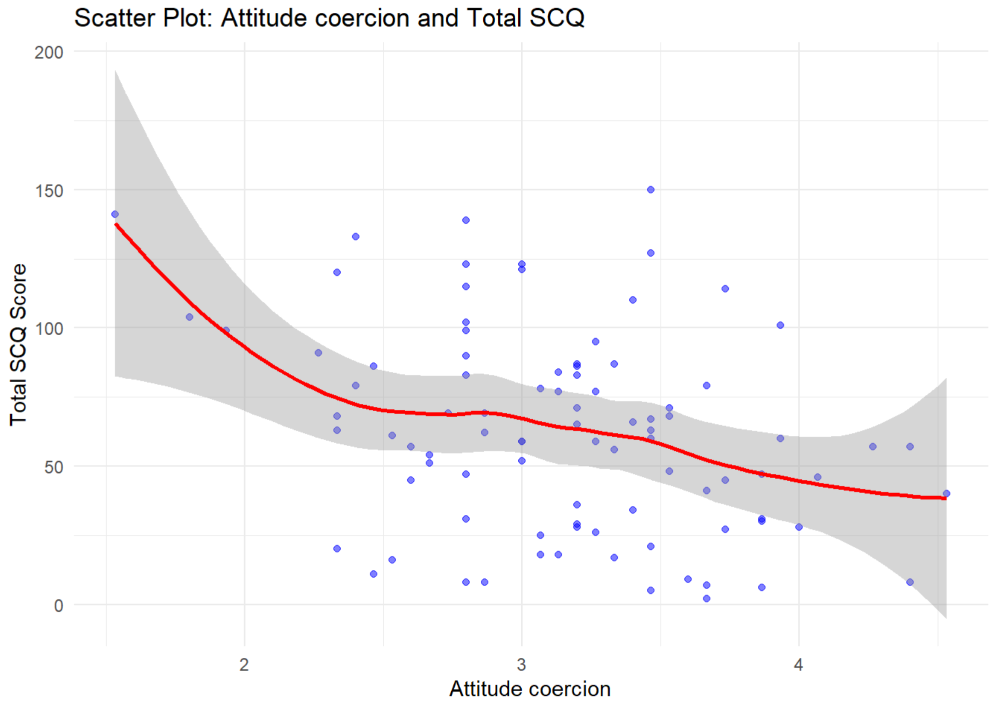

SACS subscale: Coercion as care and security

Grey shading represents the 95% confidence interval around the red trend line.

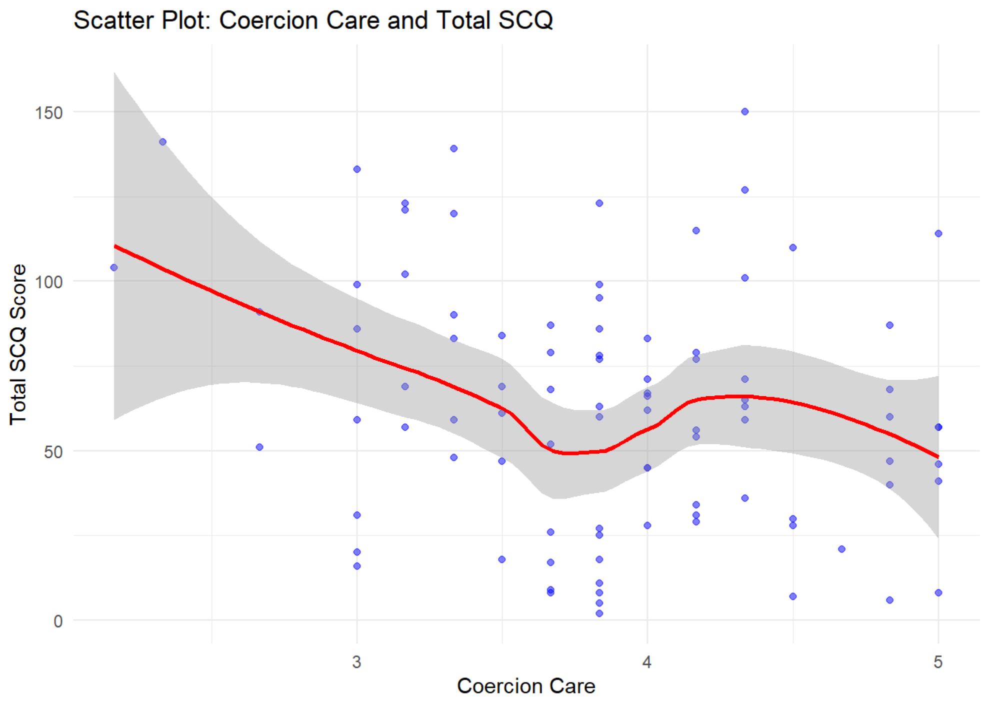

SACS subscale: Coercion as treatment

Grey shading represents the 95% confidence interval around the red trend line.

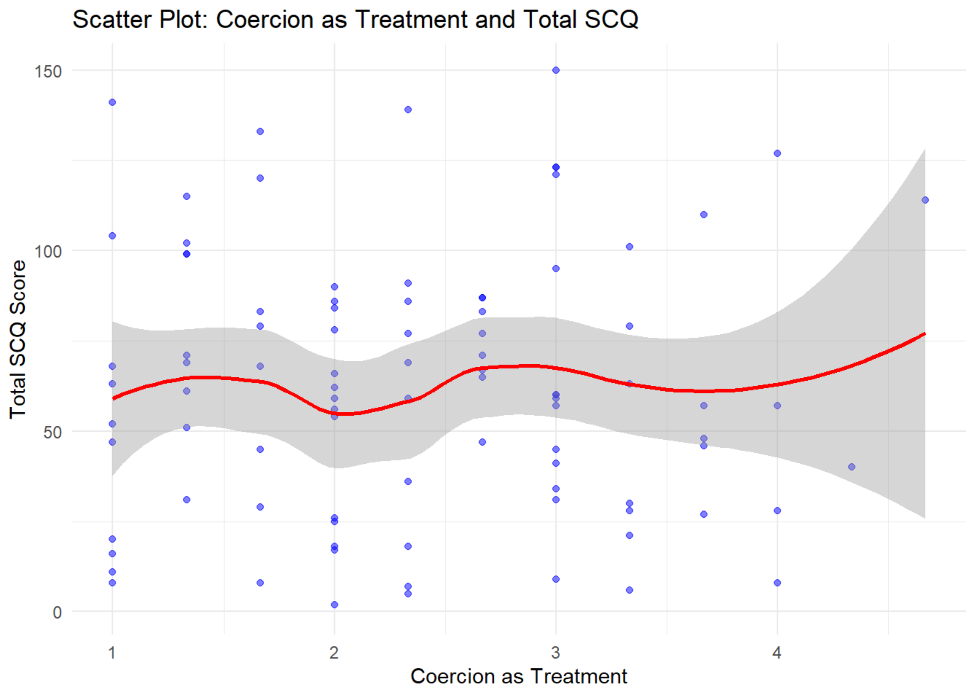

SACS subscale: *Coercion as offending (inverted)*

Grey shading represents the 95% confidence interval around the red trend line.

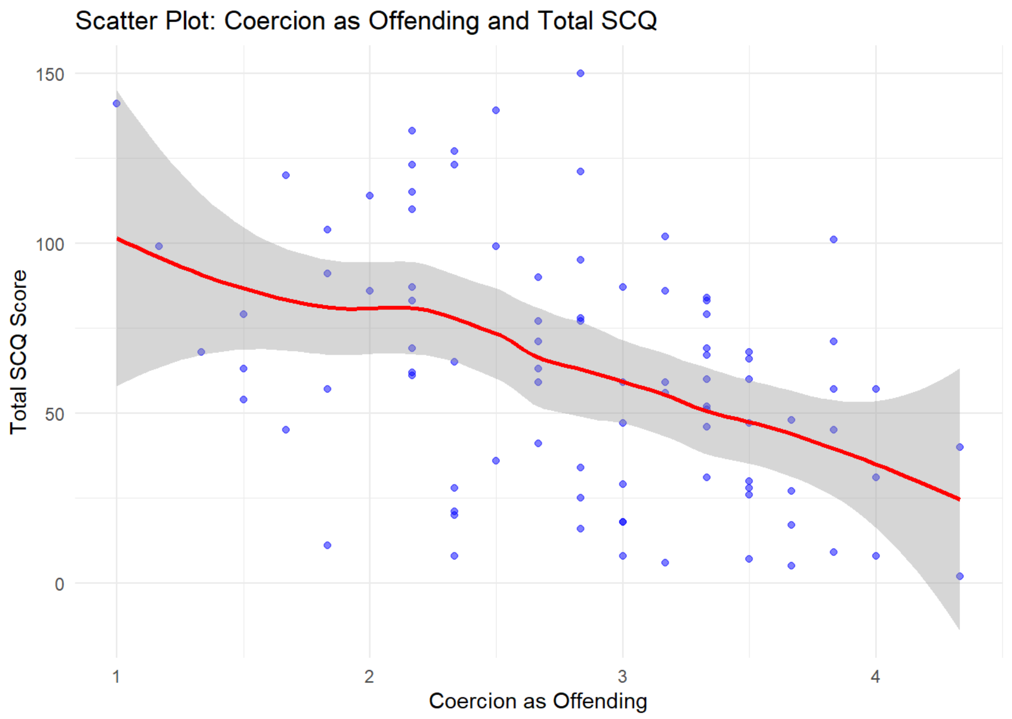

SACS-based value incongruency (perceived organizational minus personal)

Grey shading represents the 95% confidence interval around the red trend line.

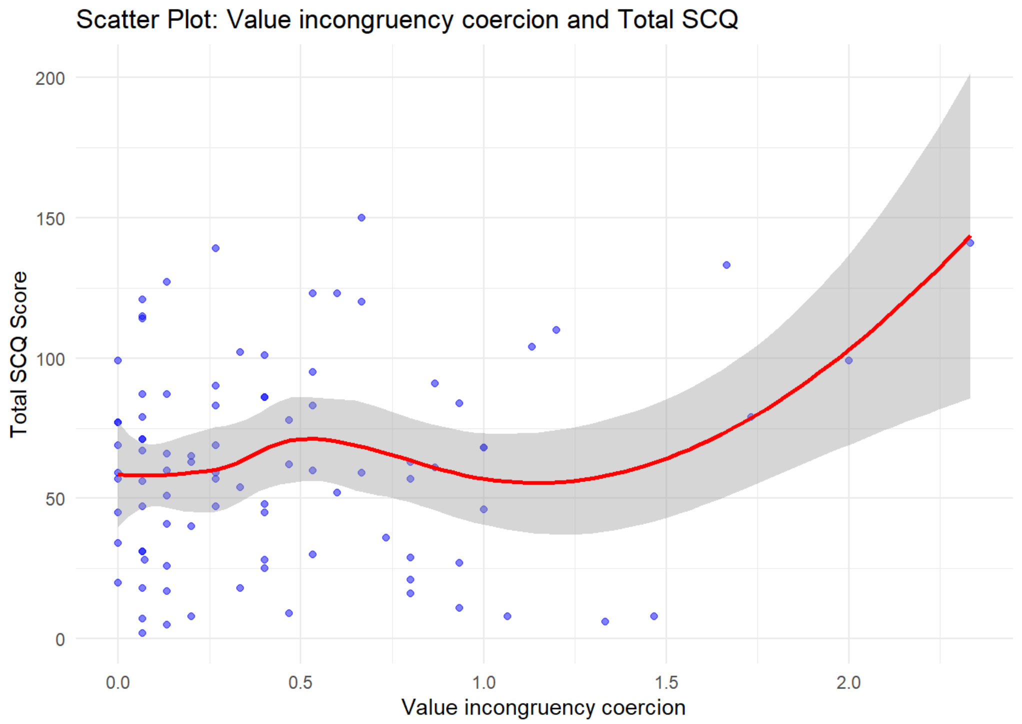

Moral distress by perceived frequency coercive measures

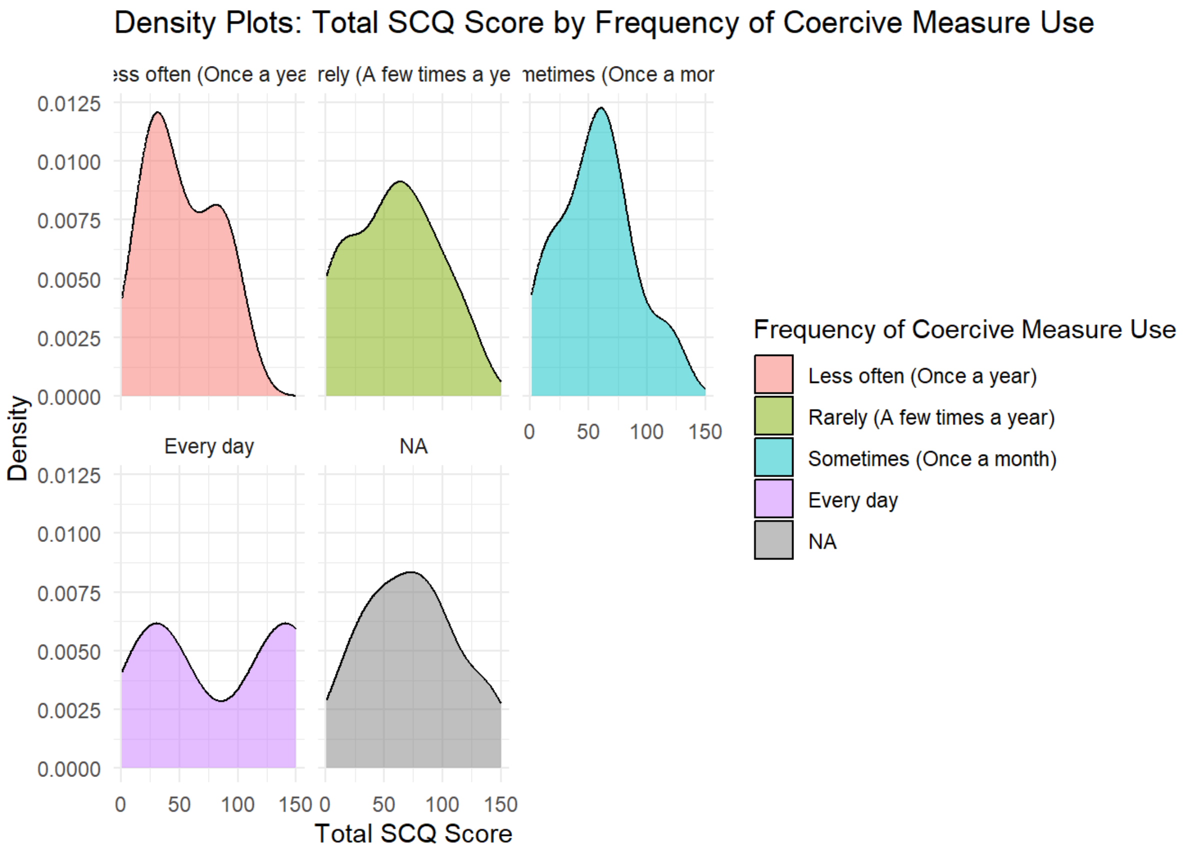

References

1. Braun V, Clarke V. Using thematic analysis in psychology. Qual Res Psychol. 2006 Jan;3(2):77–101.

2. Braun V, Clarke V. Reflecting on reflexive thematic analysis. Qual Res Sport Exerc Health. 2019 Aug 8;11(4):589–97.

3. Tavakol M, Dennick R. Making sense of Cronbach's alpha. Int J Med Educ. 2011 Jun 27;2:53–5.

Questionnaire

This is an English translation of the original Swedish questionnaire.
Due to copyright restrictions the SCQ, the SACS, and the DSCQ are not included here.

Moral Distress Among Staff in Inpatient Child and Adolescent Psychiatry in Sweden

Information and consent

Thank you for your interest in participating in our study!

What is this study about, and why should I participate?

Currently, staff turnover is high in child and adolescent psychiatry, especially in inpatient care (Psykiatrin i siffror, SKR 2021). To improve interventions and the situation for both staff and patients, we need to understand how staff are affected by providing care to complex and severely ill patients in inpatient child and adolescent psychiatry.

We are conducting a survey study targeting all staff working in inpatient child and adolescent psychiatry in Sweden. The questions focus on staff experiences of moral distress in their work with children and adolescents with psychiatric issues. Moral distress is a concept primarily used in work involving other people. It is usually defined as the stress that arises when staff— due to workload, situations, or other factors — feel that they cannot work according to their desired ethical principles.

We are targeting all clinical staff working in inpatient child and adolescent psychiatry, who encounter patients at the ward in their daily work. We aim to gain a comprehensive understanding and welcome responses from all clinically active staff, regardless of profession or employment form (temporary staff, part-time, permanent, night shifts). Hearing your perspective can contribute to a better understanding of what impacts staff in their work with this patient group.

The research organization responsible for this project is Karolinska Institutet.

How does the project work?

If you choose to participate in the study, you will answer a survey. We estimate that it will take between 10-15 minutes to complete the survey.

Potential consequences and risks of participating

Benefits of participating in the study

- Your experiences and opinions can contribute to an increased understanding of factors that affect staff in inpatient child and adolescent psychiatry.
- This understanding can enable improvements in the work environment.
- In the long term, an improved work environment for staff can promote the care of children and adolescents.

Disadvantages of participating in the study

- It takes up your time.

Consent

I have read the above information and received information on who to contact for questions. I consent to participate in the study. I also consent to the information I provide being handled as described above.

Demographic information

Hi!
We want to remind you that the survey is anonymous, and results will be presented at the group level.

1. **What gender do you identify with?**
    - Woman
    - Man
    - Specify your own
2. **If you chose the option "Specify your own," you can provide your gender identity here:**

3. **How long have you worked in inpatient child and adolescent psychiatry?**
    - Less than six months
    - 1–2 years
    - 3–5 years
    - 6–8 years
    - 8–10 years
    - More than 10 years

4. **How old are you?** (Provide age in full years)

5. **Which region do you work in?**
    - Region Blekinge
    - Region Halland
    - Region Jämtland Härjedalen
    - Region Jönköping
    - Region Kalmar

- Region Kronoberg
- Region Norrbotten
- Region Skåne
- Region Stockholm
- Region Sörmland
- Region Uppsala
- Region Värmland
- Region Västerbotten
- Region Västernorrland
- Region Västmanland
- Region Västra Götaland
- Region Örebro
- Region Östergötland
- Region Dalarna
- Region Gotland
- Region Gävleborg

6. **What is your role in inpatient care?**
    - Psychiatric aid
    - Psychologist
    - Social worker
    - Occupational therapist
    - Physiotherapist
    - Therapist
    - Physician
    - Nurse
    - Other staff
7. **If you chose the option "Other staff," please describe your role:**

[The Stress of Conscience Questionnaire (SCQ)]

[The Staff Attitude to Coercion Scale (SACS)]: individual rating

[The Staff Attitude to Coercion Scale (SACS)]: organisational rating

[Demand Support Control Questionnaire (DSCQ)]

Intention to leave job

1. **How often have you considered changing jobs in the past 12 months?**
   - Never
   - Very rarely (once a year)
   - Rarely (a few times a year)
   - Sometimes (a few times a month)
   - Often (at least once a week)
   - Every day

Free text questions

You are now on the last page of the survey. Here, you have the opportunity to elaborate on your thoughts. Otherwise, we thank you for your participation.

Moral distress is a concept primarily used in work involving people. It is usually defined as the stress that arises when staff — due to workload, situations, or other factors — feel they cannot work according to their desired ethical principles.

1. **What in your work leads to moral distress?**

2. **How does working with coercive measures affect you?**

3. **Is there anything else you would like to add?**
